# The Role of the Human Microbiome in the Pathogenesis of Pain

**DOI:** 10.3390/ijms232113267

**Published:** 2022-10-31

**Authors:** Klaudia Ustianowska, Łukasz Ustianowski, Filip Machaj, Anna Gorący, Jakub Rosik, Bartosz Szostak, Joanna Szostak, Andrzej Pawlik

**Affiliations:** 1 Department of Physiology, Pomeranian Medical University, 70-111 Szczecin, Poland; 2 Department of Medical Biology, Medical University of Warsaw, 00-575 Warsaw, Poland; 3 Independent Laboratory of Invasive Cardiology, Pomeranian Medical University, 70-111 Szczecin, Poland; 4 Department of Chemistry, The University of Chicago, Chicago, IL 60637, USA; 5 Department of Experimental and Clinical Pharmacology, Pomeranian Medical University, 70-111 Szczecin, Poland

**Keywords:** microbiome, pain, IBS, neuropathy

## Abstract

Understanding of the gut microbiome’s role in human physiology developed rapidly in recent years. Moreover, any alteration of this microenvironment could lead to a pathophysiological reaction of numerous organs. It results from the bidirectional communication of the gastrointestinal tract with the central nervous system, called the gut–brain axis. The signals in the gut–brain axis are mediated by immunological, hormonal, and neural pathways. However, it is also influenced by microorganisms in the gut. The disturbances in the gut–brain axis are associated with gastrointestinal syndromes, but recently their role in the development of different types of pain was reported. The gut microbiome could be the factor in the central sensitization of chronic pain by regulating microglia, astrocytes, and immune cells. Dysbiosis could lead to incorrect immune responses, resulting in the development of inflammatory pain such as endometriosis. Furthermore, chronic visceral pain, associated with functional gastrointestinal disorders, could result from a disruption in the gut microenvironment. Any alteration in the gut–brain axis could also trigger migraine attacks by affecting cytokine expression. Understanding the gut microbiome’s role in pain pathophysiology leads to the development of analgetic therapies targeting microorganisms. Probiotics, FODMAP diet, and fecal microbiota transplantation are reported to be beneficial in treating visceral pain.

## 1. Introduction

The gut has the most populous and diverse system of anaerobic and aerobic microorganisms in the human body [1,2,3]. It is composed mainly of bacteria. However, yeasts, archaea, or parasites living in the large area of the gastrointestinal tract often play a substantial role in this microenvironment [1,2,4,5]. The first years of life, including delivery, are crucial for the development of this complex system [6,7]. Especially at this time, selective pressure is induced by essential host and environmental factors such as breastfeeding or formula feeding, weaning age, diet, infections, and antibiotics [6,7].

**Figure 1:**
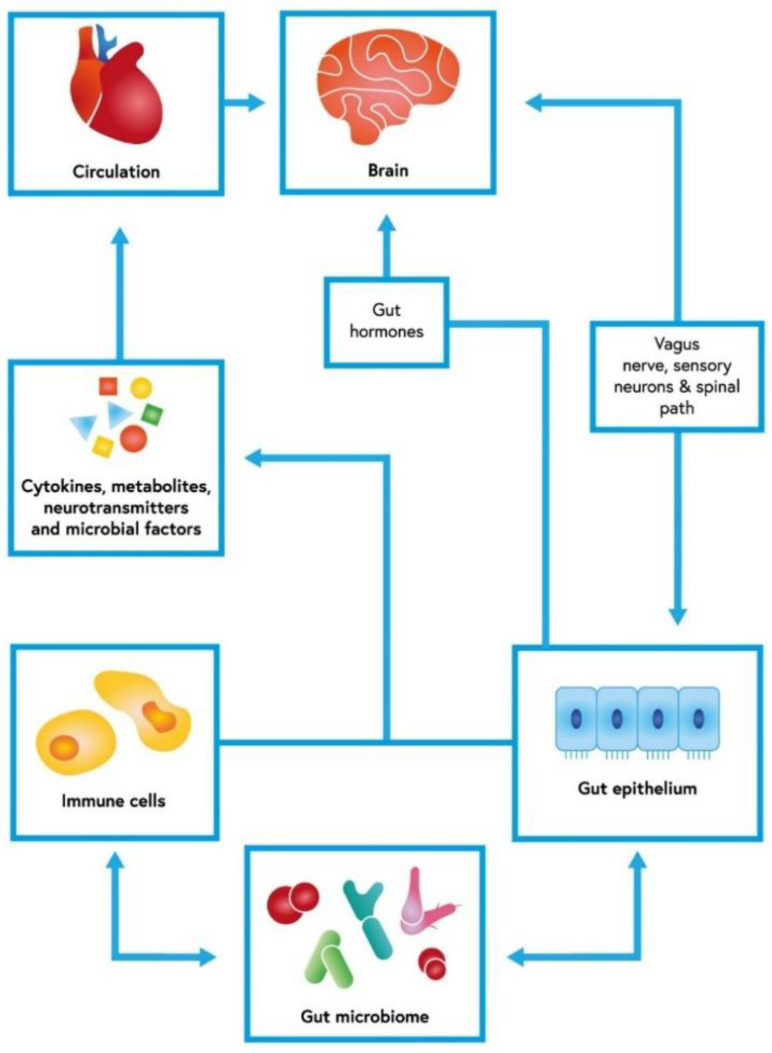
Gut–brain axis with an interconnected net of dependencies. The cerebral function could be modified by gut microbiome and its influence on gut epithelium and immune response. This bidirectional axis uses cytokines and other soluble factors, but also neuronal communication. The short-chain fatty acids (SCFAs) produced by fiber-fermenting bacteria probably have immunomodulatory functions. By binding to G-protein coupled receptors (GPR41, GPR43, and GPR109A), SCFAs exert an anti-inflammatory response in the gut mucosa [17,18].

The gut microbiota lives in homeostasis with its host. These interactions are regulated by an integral gut barrier and immune system [8,9]. The gastrointestinal tract communicates bidirectionally with the central nervous system via direct and indirect mechanisms [10]. This intricate interplay is called the gut–brain axis (GBA) [11] (Figure 1). Immunological, hormonal, and neural signals play vital roles in this interaction [10,12]. At the same time, the gastrointestinal response to central stimulation is influenced by microorganisms [11]. The microbiota participates in supplying the gut with necessary nutrients and maintaining its barrier integrity. Both terminals of the GBA use serotonin as a vital transmitter [13] Some behavioral changes regulated by serotoninergic transmission seem to depend on the microbiome [13]. Moreover, the GBA affects other systems [10,14,15,16].

Disrupted homeostasis in the GBA was first associated with gastrointestinal symptoms and disorders such as inflammatory bowel disease (IBD) or irritable bowel syndrome (IBS) [19]. Moreover, alterations in the composition of the commensal bacterial species populating the gastrointestinal tract are risk factors for a variety of diseases, including cancer [10,14,20,21,22,23]. A plant diet has an opposite effect promoting colonization of the gut by protective bacteria and inducing the production of short-chain fatty acids (SCFAs) by species such as *Faecalibacterium prausnitzii* or *Roseburia intestinalis* [24,25].

Subsequently, studies connecting the microbiota with elements of pain pathogenesis were performed. SCFAs are microbial metabolites that affect T-regulatory cells controlling inflammation [26]. Microorganisms produce neurotransmitters that, together with ingested nutrients, stimulate enteroendocrine cells to produce multiple hormones [27,28]. There is growing evidence relating the microbiome to stress, anxiety, neurological diseases, and depression [29,30,31]. Brain functions affected by microorganisms might augment nociceptive transmission [32,33,34,35].

Initiation of pain transmission is induced by nociceptors, which convert noxious stimuli into nerve impulses [36,37]. Then, the signal is modulated by multiple neurons of different types and functions or non-neuronal cells such as glia [36,37,38,39]. Nevertheless, sustained pain depends on emotional or cognitive experience [36,40]. It is regulated peripherally and centrally by substances whose production is affected by the microbiome. Pain should serve as protection from tissue damage [37]. Nonetheless, chronic pain leads to a lower quality of life [32,41]. Thus, a better understanding of its mechanism is crucial to improving the lives of millions of people worldwide. Moreover, targeting the gut microbiota seems to be a promising novel therapeutic approach for pain management.

As the aforementioned processes continue to receive increasing attention, we addressed the role of the gut microbiota in pain regulation and discussed the possibility of pain therapy by targeting the gut microbiota. In this narrative review, we collected results from in vitro and in vivo studies on the association between the GBA, pain, and its management.

## 2. Neuropathic Pain and Central Mechanisms of Pain Regulation

Neuropathic pain occurs as a result of nerve-damaging trauma or somatosensory nervous system disease, including its central and peripheral components [42]. Various conditions, such as diabetes, alcoholism, hypothyroidism, or spinal stenosis, contribute to the development of neuropathic symptoms [42]. This type of pain manifests as abnormal sensations usually felt by patients for the first time. They perceive areas of skin with a sensory deficit, paraesthesia, either spontaneous or evoked pain and thermal or mechanical hypersensitivity [42]. Some drugs used in chemotherapy treatment, such as platinum, vincristine, or toxoids, may cause chemotherapy-induced peripheral neuropathy (CIPN) [43]. Over 30% of patients fighting cancer suffer from such severe CIPN-related pain that they are not receiving sufficient treatment dosages [44].

**Figure 2:**
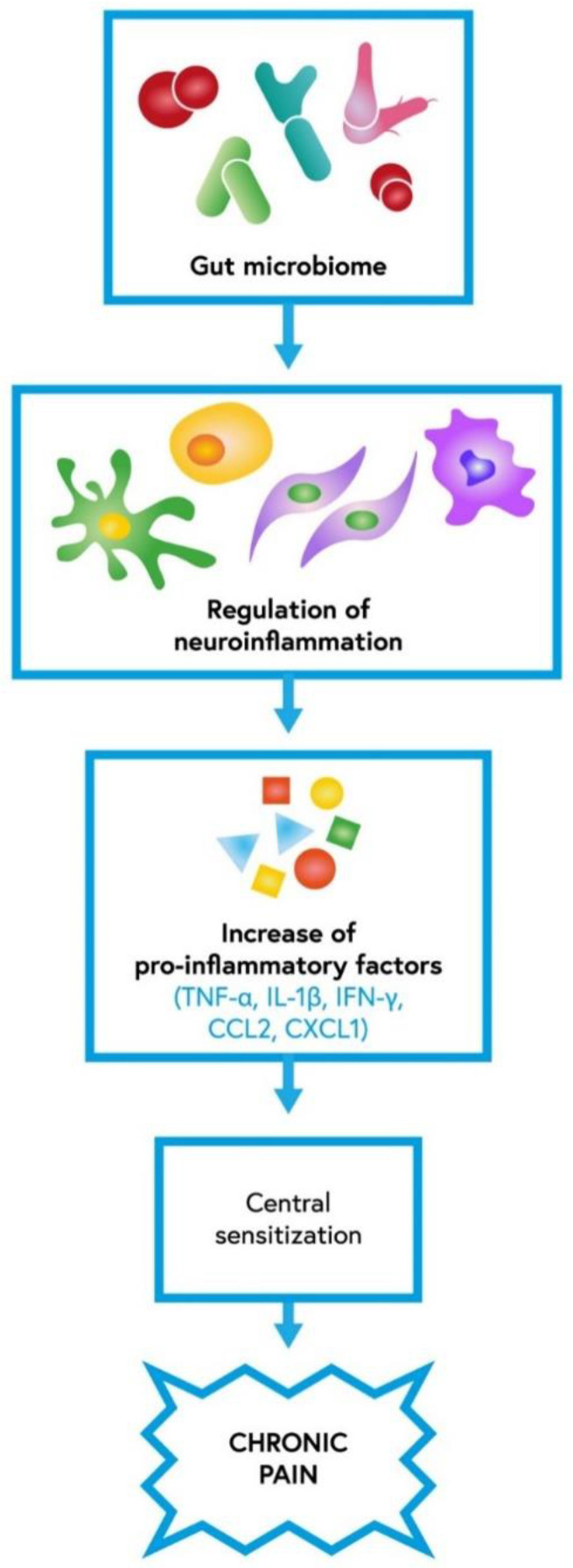
The role of gut microbiota in neuroinflammation which contributes to central sensitization underlying chronic pain; IL-1β—interleukin-1β, IFN-γ—interferon-γ, TNF-α—tumor necrosis factor-α, CCL2—C–C motif chemokine ligand 2, CXCL1—C-X-C motif chemokine 1 [12].

The gastrointestinal tract consists of various microorganisms, which are reported to play a significant role in neuroinflammatory responses. Neuroimmune activation is considered one of the primary mechanisms determining the central sensitization of chronic pain. It was shown in recent studies that the periphery, including gastrointestinal cells, might arouse brain cells [45]. The gut microbiota particularly regulates microglial function [46]. By affecting the activity of different cells, such as astrocytes, endothelial cells, microglia, monocytes, macrophages, pericytes and T-cells, the gut microbiota may regulate neuroinflammation (Figure 2). When those cells are activated, they start to produce multiple pro-inflammatory mediators such as C–C motif chemokine ligand 2 (CCL2 or MCP-1), CXCL-1, interleukin-1β (IL-1β), interferon-γ (IFN-γ), MMP-2/9, and tumor necrosis factor-α (TNF-α) [12]. Cytokines and chemokines secreted by microglia or astrocytes influence synaptic neurotransmission by increasing glutamate and decreasing gamma-amino-butyric acid (GABA) levels, resulting in pain hypersensitivity [47,48]. Taking all the data under consideration, the gut microbiota can play a major role in central sensitization underlying chronic pain associated with neuroinflammation; hence, it may contribute to the development of diverse neurological diseases [49]. Ding et al., in their article, examined the influence of the gut microbiota on neuropathic pain in chronic-constriction injury of the sciatic nerve (CCI) and whether it is associated with T-cell immune responses. CCI is an animal model widely used to represent neuropathic pain. The study showed that the gut microbiota, via modulation of both pro- and anti-inflammatory T-cell responses, induces the development of neuropathic pain. Moreover, the gut microbiota also has an impact on nociceptive behavior in sciatic nerve CCI. The study found that changes in the gut microbiota caused by the administration of oral antibiotics reduced CCI neuropathic pain. It manifested as weakened mechanical allodynia and thermal hyperalgesia [50]. Another study reported that the gut microbiota might lead to peripheral nerve trauma-induced neuropathic pain. Yang et al. showed that rats with spared nerve injury (SNI) and gut microbial dysbiosis might be prone to neuropathic pain and depression-like phenotypes, including anhedonia [46]. By contrast, in the study by Huang et al. in rat models, no significant association between oral probiotics such as *L. reuteri* LR06 or *Bifidobacterium* BL5b and anti-nociceptive effects on CCI-induced neuropathic pain was demonstrated [51]. Recent studies showed that the gut microbiota is involved in the pathogenesis of CIPN pain and modifies the effects of chemotherapeutics on tumor growth [52,53]. Shen et al. found that the gut microbiota takes part in the evolution of mechanical hyperalgesia induced by chemotherapy. In their study, mice after antibiotic treatment and germ-free mice both experienced reduced mechanical hyperalgesia after oxaliplatin administration. Moreover, restoration of the germ-free mouse microbiota revoked the protective effect [54]. Another study reported that neuropathic pain induced by paclitaxel therapy might be relieved with a DSF probiotic (high concentration of *L. plantarum*, *S. thermophilus*, *B. breve*, *L. paracasei*, *L. delbrueckii*, *L. acidophilus*, *B. longum*, *B. infantis*). Castelli et al. implied that the use of a probiotic as an adjuvant during chemotherapy might be beneficial in counteracting pain associated with CINP [55].

## 3. Inflammation and Inflammatory Pain

### 3.1. Endometriosis

Dysbiosis in the GI tract disrupts immune function, which leads to the elevation of inflammatory cytokines and alteration of immune cell profiles. Those factors may play a role in the connection between the GI tract and endometriosis, as both have a high prevalence in patients [56]. As the GI tract possesses an organized lymphoid structure with many immune cells, the gut microbiota stimulates its growth and function, as shown in a study by Hooper et al. [57]. They further showed that dysbiosis alters the composition of immune cells, triggering inflammation [56]. In the case of the vaginal microbiota, it has been shown that a non-Lactobacillus-dominant (NLD) microbiota is associated with overgrowth of pathogenic bacteria, causing bacterial vaginosis. This may decrease reproductive potency, and a vaginal microbiota rich in *Gardnerella*, *Prevotella*, and *Bacteroides* sp. may increase the risk of endometriosis or pelvic inflammatory disease (PID) [58,59,60,61,62]. In recent years, it was also discovered that the uterus, previously thought to be a sterile environment, has its own microbiota. A healthy woman’s microbiota consists primarily of *Firmicutes*, *Bacteroides*, *Proteobacteria*, and *Actinobacteria*, according to a study by Baker et al., and a review by Moreno et al. identified the five most represented genera in the endometrial microbiota [62,63,64].

Ata et al. studied women with stage III/IV endometriosis and compared their microbiota from the gut, cervix, and vagina to that of a control group of healthy women. The cervical microbiota of women with endometriosis had an increased number of pathogenic species, and stool samples had higher *Shigella* and *Escherichia* concentrations [65]. Other studies also found a correlation between the increase in bacteria associated with bacterial vaginosis or opportunistic pathogens in the reproductive tract with endometriosis in women [61,65,66,67,68,69,70,71,72,73,74].

### 3.2. Chronic Pelvic Pain

Chronic pelvic pain (CPP) is a long-lasting pain that lowers quality of life, with many possible causes, such as endometriosis or chronic bacterial prostatitis [75,76]. Recent discoveries regarding the gut microbiome and visceral pain led to hypotheses about the correlation between CPP and the human microbiota. Shoskes et al. determined that patients with CPP had lower gut microbiota diversity than the control group, especially amongst *Prevotella* [77]. A study by Du et al. created a mouse model with experimental autoimmune prostatitis (EAP). EAP mice developed changes in the gut microflora, resulting in a distorted balance in Th17/Treg cells and decreased levels of short-chain fatty acids (SCFAs) in both serum and feces. Microbiota of healthy mice had notably fewer *Firmicutes*, *Nitrospirae*, or *Fusobacteria* than those with EAP. Additionally, the EAP mice had bacteria producing SCFAs, including *Bacteroides*, *Butyricicoccus*, and *Ruminococcaceae*. Changes in Th17/Treg balance were later reversed by supplementation of the SCFA propionate [78]. Their findings were consistent with other studies regarding chronic non-bacterial prostatitis [79,80]. Pelvic allodynia may also be caused by deficient lipase acyloxyacyl hydrolase (AOAH), an enzyme present in microglia. A study by Rahman-Enyart et al. suggests that AOAH plays a role in the modulation of pelvic pain, and its production is dependent on changes in the gut microbiome [81]. As new studies show, the microbiota is a crucial part of overall health, and its changes are correlated with many illnesses; however, further research is needed to make a comprehensive understanding of this topic possible.

## 4. Visceral Pain, Peripheral Mechanisms of Pain Regulation, and IBS

Visceral pain is a medical term for pain originating from the internal organs within the thorax or abdomen and is divided into acute and chronic pain. Acute visceral pain, caused by typically identifiable causes, is treated with appropriate therapeutic agents, including over the counter (OTC) medications such as non-steroidal anti-inflammatory drugs (NSAIDs) or acetaminophen, and is relatively easy to cure. On the other hand, chronic visceral pain can be difficult to treat even with opioids, and its unknown pathology led to the creation of the term functional gastrointestinal disorders (FGIDs), a collection of many disorders in pediatric and adult patients. FGID includes terms such as irritable bowel syndrome (IBS), infant colic and abdominal migraine, or functional dyspepsia. In the gastrointestinal tract (GI tract), nociceptor nerve endings are found throughout the layers of the GI tract. They respond to many stimuli from the tract and transfer them to their cell bodies in the dorsal horn of the spinal cord [82]. After being transferred to the contralateral side of the spinal cord, the signal is then transmitted to the limbic part of the brain via the spinothalamic tract. A response is then created, and a descending inhibitory circuitry is activated, causing a release of inhibitory neurotransmitters.

In recent years, scientists studied how the microbiome of the GI tract may influence the visceral pain response. The microbial population of a person stabilizes after the first 3 years of life and from then on is relatively stable [82]. Its greatest changes are noticed during disease states; however, while disorders affecting the GI tract are the more obvious causes, GI tract dysbiosis has been observed in many other illnesses. Non-intestinal disorders, such as obesity, allergy, asthma, or autoimmune diseases can also be a factor [82,83,84,85]. Additionally, the use of broad-spectrum antibiotic treatment changes the gut microbiota, and using such antibiotics without strong clinical purpose may become a factor in IBS. In a study by Vicentini et al., mice treated with broad-spectrum antibiotics showed effects on the structure and function of the GI tract, resulting in the loss of enteric neurons in enteric plexuses. Post-treatment supplementation of short-chain fatty acids (SCFAs), naturally produced by a healthy gut microbiome, restored neuronal loss in both submucosal and myenteric plexuses [86]. Similarly, a study by De Palma et al. focused on replicating IBS dysbiosis in rats. With fecal microbiota transplant in rats, visceral hypersensitivity increased when compared to gnotobiotic rats receiving a healthy microbiota, suggesting a link between IBS-associated hypersensitivity and the intestinal microbiota [87,88].

### 4.1. IBS

The influence of the gut microbiota and its dysbiosis on the pathophysiology of IBS was investigated in many studies that compared differences in the GI tract microbiome between IBS patients and controls [89,90,91,92]. Those studies showed that the intestinal microbiome of IBS patients had reduced amounts of *Bacteroides*, *Prevotella*, and *Parabacteroides* sp. Noor et al. and Maccaferi et al. showed that IBS patients had an increased population of *Bacillus*, *Bifidobacteria*, *Lactobacillus*, *Clostridium*, and *Eubacterium rectale* [93,94,95]. Those studies led to research about probiotic intervention and its benefits for IBS patients. In a study by Sisson et al., Symprove, a probiotic containing three *Lactobacillus* types and one Enterococcus, was shown to improve symptom severity in IBS [96]. In another study by Guglielmetti et al., *Bifidobacterium bifidum* MIMBb75 alleviated IBS symptomology by decreasing pain, discomfort, digestive upset, or bloating [97]. Further studies on probiotics for IBS presented another bacterial species with a positive influence on the relief of IBS symptoms [13,98]. Butyrate producers such as *Faecalibacterium* sp. have an anti-inflammatory impact on the GI tract, and *F. prausnitzii* is a source of serine protease that was shown to have anti-nociceptive activity by decreasing the excitability of dorsal root ganglia neurons [99,100,101,102]. Unknown IBS pathophysiology led to the creation of the term ‘psychobiotics’, referring to probiotics and bacterial metabolites that signal directly to the brain. In a randomized controlled trial (RCT) of 44 adults with IBS, patients were treated with *Bifidobacterium longum* NCC3001. Patients showed a significant reduction in depression and an increase in quality of life with no change in IBS symptom severity or the fecal microbiota profile. This suggested that there is some direct signaling of *B. longum* metabolites to the central nervous system (CNS) [103].

### 4.2. Peripheral Mechanism of Pain Regulation

The enteric nervous system (ENS) is formed by about 200–600 million neurons and is often referred to as the ‘second brain’. This network plays a part in maintaining GI tract function and reaches the lamina propria of the mucosa. ENS neurons form the subserous, myenteric, and submucosal plexuses and carry impulses to and from the brain. Intrinsic primary afferent neurons (IPANs) initiate secretory, motor, and vasomotor reactions from stimuli within the mucosa and from the central nervous system (CNS) [104]. Enteric sensory neurons receive the information through neurotransmitters and hormones released by enteroendocrine (EEC) and enterochromaffin (EC) enteric cells.

Enteric hormones such as serotonin (5-HT), glucagon-like peptide 1 (GLP-1), or peptide YY (PYY) are thought to have an impact on visceral pain and its management [104]. 5-HT excreted by EC cells activates receptors on EC cells and extrinsic primary afferent nerve (EPAN) terminals. This triggers enteric reflexes such as secretion, peristalsis, and perception of pain and inflammation [105,106,107].

Microbes in the GI tract microbiome can synthesize various neurotransmitters and metabolites involved in gut–brain communication, as shown in recent studies [108,109,110,111]. This includes SCFAs, tryptophan metabolites, GABA, dopamine, and noradrenaline [104]. One of the SCFAs, butyrate, was proposed as an agent with an indirect effect on regulating inflammatory visceral pain. Its injection in rat and mouse brains stimulated the production of brain-derived neurotrophic factor (BDNF), which favors neurogenesis, memory formation, and mood stabilization [112,113,114].

Bacteria such as *Escherichia*, *Fusobacterium*, *Prevotella*, *Enterococcus casseliflavus*, or *Bacteroides* can produce tryptophan, which later passes the blood–brain barrier (BBB), influencing serotoninergic neurotransmission in the CNS. In a study by Agus et al., it was shown that during gut inflammation, an increase in tryptophan conversion to kynurenine may be responsible for the development of anxiety and mood shifts [115]. During inflammation, there is an enhanced plasma level of kynurenine, which may favor its passage through the BBB and later metabolism into kynurenic acid (KynA) and quinolinic acid (QuiA), the latter of which is described as a neurotoxic agent [111].

Another microbial product, glutamate, is produced by certain microbial strains in the healthy GI tract [116,117,118,119]. It is a major neurotransmitter in the CNS and acts as a neuroactive molecule. A recent study suggested that glutamate may also regulate gut sensory and motor functions via receptors in the ENS [120,121]. During stress-induced dysbiosis, glutamate receptor expression is altered. In antibiotic-treated mice with dysbiosis, there were decreased levels of hippocampal NMDA and BDNF, which were later restored by prebiotic treatment [31,122,123,124].

GABA is an important neurotransmitter in the brain. Bravo et al. studied its role in pain management and suggested that GABA can inhibit visceral hypersensitivity, altering abdominal pain [125]. Oral administration of *Lactobacillus rhamnosus* in mice increased GABA levels in the CNS. Additionally, in a study by Perez-Berezo et al., administration of the *E. coli* Nissle 1917 (EcN) strain showed an increase in analgesic lipopeptide production, activation of GABA receptors on IPANs, and inhibition of visceral hypersensitivity [126].

## 5. Headache and Its Association with Drugs

Headache is one of the most frequently reported symptoms [127], and various types have been described. Primary headaches can be divided into four groups: migraine, tension headache, trigeminal autonomic cephalgia, and other primary headache disorders [127]. Migraine, a neurological disorder characterized by headache, nausea, vomiting, and photophobia or phonophobia [128,129], is one of the most common types of headaches [17]. The hemicrania occurs due to hypothalamus activation and further pituitary adenylate cyclase-activating polypeptide (PACAP) secretion, which is responsible for vasodilatation [17]. Moreover, migraine is related to GI illnesses, which include celiac syndrome, irritable bowel syndrome, or infection by *Helicobacter pylori* [12,30]. There is also an association between the gut microbiome and the pathogenesis of migraine. The gut–brain axis triggers the migraine attack through pro-inflammatory factors, gut microbiome composition, neuropeptides, serotonin pathways, stress hormones, and nutritional substances. The physical or psychological stress factors may lead to gut microbiome changes such as dysbiosis [30]. This, in turn, causes an increase in calcitonin gene-related peptide (CGRP) secretion [17], which is correlated with migraine symptoms and has antibacterial effect on strains such as *E. coli*, *E. faecalis*, and *L. acidophilus* [17,30]. This particular type of headache is associated with pro-inflammatory factors. During migraine attacks, increased secretion of serum cytokines such as IL-1b, IL-6, IL-8, and TNF-a was observed. Moreover, Arzani et al. reported that in germ-free mice, the hypernociception induced by inflammatory mediators is reduced [30]. These could result from increased expression of IL-10 in germ-free mouse models [130]. This cytokine is an important regulator of inflammatory responsiveness [130]. These lines of evidence emphasize the importance of the gut microbiome in migraine and have prompted research on whether probiotic supplementation is a beneficial therapy for the condition [12]. The data on the efficacy of probiotic supplementation in migraine are incoherent. Sensenig et al. showed that most patients who were given probiotics, such as *L. bulgaricus*, *L. acidophilus*, *E. faecium*, and *B. bifidum*, reported an improvement in quality of life [131]. By contrast, another study showed no significant differences between a group of patients who suffered from migraine and were supplemented with probiotics and the one that was not supplemented with probiotics [12,132].

To summarize, the association between the gut microbiome and migraine is clear. Studies show not only a correlation in pathogenesis but also a possible way of treating migraine with probiotics. However, there is still a lot to be discovered [12].

### Opioid Tolerance

Opioids are known for their anti-nociceptive, anti-tussive and anti-diarrheal properties. They are the major drugs used in cancer and post-surgical pain treatment [133], although their severe side effects, such as tolerance, dependence, emesis, or constipation, lead to significant restrictions in their use [12]. GI symptoms associated with these drugs are known as opioid bowel dysfunction (OBD) and are the result of the stimulation of opioid receptors in the GI tract [134]. The research shows that chronic use of opioids may result in dysbiosis [12,135], damage to the gut barrier, bacterial translocation, and secretion of pro-inflammatory factors. Opioid tolerance was associated with a lack of *Bifidobacteria* and *Lactobacillaceae* in mice [12,25]. The enteric glia are responsible for the proper functioning of the GI tract [12]. Furthermore, they are also relevant to the development of the ectypal inflammatory reaction to long-term use of opioid drugs [136]. The bacterial product bacterial lipopolysaccharide (LPS) was reported to be associated with the production of pro-inflammatory cytokines during long-term opioid treatment [136]. Due to the chronic use of morphine, we can observe increased activity in enteric glia of the P2X receptor [12,136], a calcium-permeable ion channel activated by ATP and associated with cytokine secretion by enteric glia [25]. This leads to an enhanced inflammatory reaction [25]. LPS is also related to the intensified expression of connexin 43 (Cx43), a gap junction protein that mediates the secretion of ATP [136]. Cx43 can be blocked by non-specific connexin inhibitor (CBX), which results in a decreased inflammatory response [136].

Another study showed that administration of broad-spectrum antibiotics prevents GI side effects and tolerance to opioid-related drugs with long-term use of morphine [137]. Analgesic tolerance can be avoided by oral vancomycin due to its active properties against Gram-positive bacteria, the translocation of which is significant in the tolerance process [12,25,137]. Furthermore, germ-free mice have reduced morphine tolerance, which can be reclaimed by gut microbiome reconstitution [138]. In addition, opioid tolerance can be a result of the inactivation of tetrodotoxin-resistant (TTX-R) Na+ channels in dorsal root ganglia (DGR) neurons, which can be reversed by oral vancomycin administration [139]. In conclusion, the above-described studies prove the importance of the gut microbiota in opioid tolerance occurrence. They show the role of the gut flora in the genesis of morphine tolerance and indicate how the side effects of opioid drug use may enhance the entire process.

## 6. The Gut Microbiota as a Therapeutic Target in Chronic Pain

### 6.1. Probiotics and Prebiotics

Probiotics are living microorganisms that can provide health benefits to the host [140]. A growing body of research supports the thesis that probiotics are effective in modifying the balance of the gut microbiota [141,142]. Some of their proven beneficial effects include improved digestion, boosted immunity, and decreased cholesterol levels [143]. Some of the more recent studies suggest that probiotics might be effective in alleviating the symptoms of chronic intestinal disorders, such as Crohn’s disease [144].

Several preclinical animal studies have demonstrated the beneficial effects of probiotics on visceral pain [145,146,147]. In multiple studies, probiotics exerted beneficial effects on visceral hypersensitivity. In rats, probiotic VSL#3 decreased visceral hypersensitivity potentially through the mast cell-PAR2-TRPV1 pathway, which then affects the release of potent mediators that affect the enteric nerves and smooth muscles [145]. Moreover, supplementation with *Clostridium butyricum*, a commensal bacterium, may inhibit colonic inflammation in a mouse model of IBS through its action on nod-like receptor pyrin domain-containing protein 6 [146]. In a similar model, *Roseburia hominis* alleviated visceral hypersensitivity and prevented the expression of occludin from decreasing [147]. Moreover, in rats, *B. infantis* 35624 significantly reduced visceral pain, suggesting that it may be effective in treating symptoms of IBS [148].

Several human studies have also revealed the benefits of using probiotics for chronic pain. A randomized, double-blind study on 101 pediatric patients suffering from IBS (NCT01180556) revealed that a 4-week supplementation of *L. reuteri* DSM 17938 reduced both the frequency and the intensity of abdominal pain in children [149]. Moreover, a probiotic mixture of *Bifidobacterium infantis* M-63, breve M-16V, and longum BB536 (NCT02566876) was successful in attenuating the symptoms of abdominal pain in IBS but not in functional dyspepsia. Likewise, the intervention group noted the markedly higher quality of life improvement in comparison with a placebo (48% vs. 17%, *p* = 0.001) [150]. A 2009 review by Newlove-Delgado et al. retrospectively investigating the use of probiotics in children with recurrent abdominal pain suggested that those preparations are likely to improve pain symptoms in the short term, that is, up to 3 months (OR = 1.63; 95% CI = 1.07–2.47) [151]. By contrast, a randomized, placebo-controlled trial by Spiller et al. failed to identify any clinical benefit, including intestinal pain and discomfort, of *S. cerevisiae* I-3856 supplementation at a dose of 1000 mg per day, in comparison to a placebo [152].

Prebiotics are fibers and other non-digestible ingredients that benefit the host by selectively boosting the growth and activity of select microorganisms in the colon, mainly lactobacilli and bifidobacteria. They are considered either as an addition to probiotics or an alternative to them. Several pre-clinical dissertations have emerged underlining the beneficial role of prebiotics in terms of attenuating chronic pain, such as PDX/GOS reducing chronic visceral pain induced by intracolonic zymosan injection in rats [153]. In human studies, a prebiotic galacto-oligosaccharide mixture supplemented for 2 weeks reduced abdominal pain associated with GI disorders in adults. The treatment arm reported significantly lower scores for bloating, flatulence, and pain. However, there was no improvement in quality of life throughout the study [154]. Lastly, a study on the symbiotic containing *Bacillus coagulans* on 88 pediatric patients showed a reduction of abdominal pain that was present after treatment (60% vs. 39.5%, *p* = 0.044) but not after 12 weeks of follow-up [155].

### 6.2. FODMAP Diet

Recent studies have demonstrated that functional GI symptoms can be induced by colonic gas production in patients with visceral hypersensitivity. In those patients, short-chain fermentable carbohydrates increase small intestinal water volume, resulting in increased colonic gas production. Therefore, dietary restriction of short-chain fermentable carbohydrates (low-FODMAP diet) should theoretically ameliorate the symptoms of IBS. In pre-clinical studies, the low-FODMAP diet (LFD) altered the gut microbial composition, resulting in reduced fecal lipopolysaccharide of Gram-negative bacteria. In contrast to a high-FODMAP diet, there is a significant reduction of *Akkermancia muciniphila* and *Actinobacteria* [156]. Therefore, it could be beneficial in reducing gut mucosal inflammation and restoring the barrier function of the gut, ultimately leading to the alleviation of visceral pain [156].

In a clinical setting, the FODMAP diet has led to a reduction in IBS severity, with decreased frequency of pain episodes (*p* < 0.01) and increased quality of life [157]. In another study by Pedersen et al., LFD resulted in a greater reduction of disease severity but no improvement in quality of life [158]. In a double-blind, placebo-controlled trial on 40 patients with IBS by Hustoft et al., LFD with fructans lowered the severity of nausea, vomiting, and flatulence [159]. Overall, up to 86% of IBS patients improve clinically in terms of GI symptoms, as well as abdominal pain, bloating, and constipation, while following the diet [160].

### 6.3. Fecal Microbiota Transplantation

Fecal microbiota transplantation (FMT) involves the transfer of microbial flora from a healthy donor stool to the recipient’s intestinal tract to normalize the target intestinal microbiota composition and function. One of the most notable examples of the use of FMT in clinical practice is *Clostridium difficile* infection (CDI), which often occurs in patients whose microbiota has been suppressed by prolonged antibiotic therapy. Recent evidence suggests that the gut microbiota composition is linked to the occurrence of abdominal pain and its frequency, duration, and intensity in the general population [161]. Moreover, in an animal model of colitis, FMT administration to control rats resulted in long-lasting visceral hypersensitivity [162]. Several mechanisms have been proposed through which FMT might affect chronic pain, including competition with pathogenic bacteria, protection of the intestinal barrier, or stimulation of the intestinal immune system [163]. An open-label study on FMT in humans with IBS showed marked improvement in abdominal pain that was associated with the abundance of *Akkermansia muciniphila* [164].

Moreover, allogenic FMT resulted in a significant decrease in symptoms of IBS (*p* = 0.02), which was not present in the autologous FMT group (*p* = 0.16) [165]. Furthermore, a metagenomic sequencing study revealed that following FMT the taxonomic profile of the recipient shifts towards a donor-like profile, inducing long-term changes in the gut microbiota, which mirror the clinical effect of the treatment [166].

In order to perform successful FMT, several criteria must be met. Firstly, donor selection should be strict, excluding those at risk of harboring an infectious agent. Moreover, recipients must not receive major immunosuppressive therapy, or suffer from serious comorbidities that would put them at risk [167]. While FMT is relatively safe, some of the studies suggest its potential drawbacks. One study suggested that FMT might be associated with diarrhea, abdominal cramping, belching, and nausea within 3 h post-FMT [168]. Moreover, there exists a possibility of development of long-term adverse effects due to alteration of the gut microbiota. More long-term, follow-up studies are required to address this issue [169].

## 7. Conclusions

In recent years, numerous studies have provided data on the role of the gut microbiome and its influence on other tissues. It is known that alteration in the microbiome could be one of the factors contributing to the development of cancer and neurological, gastrointestinal, cardiovascular, and metabolic diseases. Lately, many studies have also investigated the role of the human microbiome in the pathogenesis of different types of pain (Table 1). Proper assessment and control of pain are essential for improving quality of life in many patients. Despite the availability of various pain management methods, there is still a great need for research on factors contributing to pain pathogenesis and novel therapies. Recent studies suggest that the human microbiome may be an essential component of the pathogenesis of multiple types of pain. Neuropathic pain could result from the gut microbiome’s influence on T-cell immune response, disrupting the regulation of pro- and anti-inflammatory cytokine production. Furthermore, alteration of the immune cell response and cytokine production by the gut microbiome could contribute to the development of inflammatory diseases, such as endometriosis. Chronic visceral pain remains a challenge to efficient treatment. The human microbiome contributes to the still unknown pathogenesis of FGIDs, providing a promising direction for further studies. Additionally, common symptoms such as headaches are influenced by the gut microbiome. An altered gut–brain axis could trigger a migraine. Moreover, the regulation of inflammatory mediators that contributes to migraine is disrupted by dysbiosis. The gut microbiome could also impact the efficacy of pain management, leading to opioid tolerance. The contribution of the human microbiome to the pathogenesis of multiple types of pain leads to its use as a possible target for analgesic therapies. Pro- and prebiotics are already widely used in clinical practice. They are reported to be effective in reducing chronic visceral pain and migraine. However, there is still a great need for further evaluation of their efficacy and influence on patients’ quality of life. Another approach assumes the modification of the gut microbiome with a specific diet, such as a low-FODMAP diet, which could be beneficial for patients with IBS, reducing symptoms and pain episodes. The usage of FMT is recommended in the treatment of *Clostridium difficile* infection.

Moreover, FMT is reported to efficiently reduce visceral pain among IBS patients. However, these studies have some limitations. There is a strong need for further evaluation of concepts and previous results. Additional long-term studies are required to assess the potential side effects of gut microbiota alteration. Moreover, the differences in the methodology of the studies impede the precise comparison of the results. Pittayanon et al., in their systematic review, reported concerns about deficiencies in studies’ methodology and statistical analysis [95]. The shortcomings, such as lack of data on administrated antibiotics, and differences in the microbiome evaluation methods, are reasons for inconsistency in reviewed papers.

Despite the significant development in the understanding of the human microbiome in the pathogenesis of pain, there are still many areas to be investigated. A detailed evaluation of the influence of the altered microbiome on the gut–brain axis could be a critical factor in understanding the impact of dysbiosis on several tissues and pain development [18]. The detailed characterization of the gut microbiome in chronic, visceral, or headache states and their interaction with the gut–brain axis could deliver novel insight into the pathogenesis of a different type of pain. Further molecular studies could develop novel targets for analgetic treatment that could significantly improve numerous patients’ quality of life.

## Figures and Tables

**Table 1:** Summary of novel studies that investigated the role of the human microbiome in the pathogenesis of different types of pain.

Neuropathic Pain and Central Mechanisms of Pain Regulation	Inflammation and Inflammatory Pain	Visceral Pain, Peripheral Mechanisms of Pain Regulation, and IBS	Headache and Its Association with Drugs	The Gut Microbiota as a Therapeutic Target in Chronic Pain
[46] Yang C, Fang X, Zhan G, et al. Key role of gut microbiota in anhedonia-like phenotype in rodents with neuropathic pain.	[56] Jiang I, Yong PJ, Allaire C, Bedaiwy MA. Intricate Connections between the Microbiota and Endometriosis.	[82] Moloney RD, Johnson AC, O’Mahony SM, Dinan TG, Greenwood-Van Meerveld B, Cryan JF. Stress and the Microbiota–Gut–Brain Axis in Visceral Pain: Relevance to Irritable Bowel Syndrome.	[17] Léa LT, Caula C, Moulding T, Lyles A, Wohrer D, Titomanlio L. Brain to Belly: Abdominal variants of migraine and functional abdominal pain disorders associated with migraine.	[145] Li Y-J, Dai C, Jiang M. Mechanisms of Probiotic VSL#3 in a Rat Model of Visceral Hypersensitivity Involves the Mast Cell-PAR2-TRPV1 Pathway.
[42] Baron R, Binder A, Wasner G. Neuropathic pain: diagnosis, pathophysiological mechanisms, and treatment.	[77] Shoskes DA, Wang H, Polackwich AS, Tucky B, Altemus J, Eng C. Analysis of Gut Microbiome Reveals Significant Differences between Men with Chronic Prostatitis/Chronic Pelvic Pain Syndrome and Controls.	[104] Morreale C, Bresesti I, Bosi A, Baj A, Giaroni C, Agosti M, et al. Microbiota and Pain: Save Your Gut Feeling.	[12] Guo R, Chen LH, Xing C, Liu T. Pain regulation by gut microbiota: molecular mechanisms and therapeutic potential.	[146] Zhao K, Yu L, Wang X, He Y, Lu B. Clostridium butyricum regulates visceral hypersensitivity of irritable bowel syndrome by inhibiting colonic mucous low grade inflammation through its action on NLRP6.
			[25] Santoni M, Miccini F, Battelli N. Gut microbiota, immunity and pain. Immunol	[147] Zhang J, Song L, Wang Y, Liu C, Zhang L, et al. Beneficial effect of butyrate-producing Lachnospiraceae on stress-induced visceral hypersensitivity in rats.
			[136] Bhave S, Gade A, Kang M, Hauser KF, Dewey WL, Akbarali HI. Connexin-purinergic signaling in enteric glia mediates the prolonged effect of morphine on constipation.	

## Data Availability

Not applicable.
